# Maternal food restriction during pregnancy affects offspring development and swimming performance in a placental live-bearing fish

**DOI:** 10.1242/jeb.242850

**Published:** 2022-01-20

**Authors:** Andres Hagmayer, Martin J. Lankheet, Judith Bijsterbosch, Johan L. van Leeuwen, Bart J. A. Pollux

**Affiliations:** Department of Animal Sciences, Wageningen University, 6708 WD Wageningen, The Netherlands

**Keywords:** Life-history, Matrotrophy, Placenta, Placentotrophy, Poeciliidae, Viviparity

## Abstract

How pregnant mothers allocate limited resources to different biological functions such as maintenance, somatic growth, and reproduction can have profound implications for early life development and survival of offspring. Here, we examined the effects of maternal food restriction during pregnancy on offspring in the matrotrophic (i.e. mother-nourishment throughout gestation) live-bearing fish species *Phalloptychus januarius* (Poeciliidae). We fed pregnant females with either low or high food levels for 6 weeks and quantified the consequences for offspring size and body fat at birth and 1 week after birth. We further measured fast-start escape performance of offspring at birth, as well as swimming kinematics during prey capture at 0, 2 and 7 days after birth. We found that the length of maternal food restriction during pregnancy negatively affected offspring dry mass and lean dry mass at birth, as well as body fat gain during the first week after birth. Moreover, it impacted the locomotor performance of offspring during prey capture at birth and during the first week after birth. We did not observe an effect of food restriction on fast-start escape performance of offspring. Our study suggests that matrotrophic poeciliid fish are maladapted to unpredictably fluctuating resource environments, because sudden reductions in maternal food availability during pregnancy result in smaller offspring with slower postnatal body fat gain and an inhibition of postnatal improving swimming skills during feeding, potentially leading to lower competitive abilities after birth.

## Abstract (precis)

**Highlighted Article:** Maternal food restriction during pregnancy results in smaller offspring, slower postnatal body fat gain and an inhibition of postnatal improvement of swimming skills during feeding, possibly leading to lower competitive abilities after birth.

## INTRODUCTION

How individuals allocate resources to different biological functions such as maintenance, somatic growth and reproduction is crucial for their life-history (Stearns, 1992). Different functions compete for limited resources, leading to trade-offs and a limited set of possible life-history strategies (Braendle et al., 2011).

In addition to intrinsic trade-offs and constraints, resource allocation and hence life-histories are also influenced by environmental factors, such as food availability (Boggs, 1992; Santi et al., 2020). Increased maternal food availability enables a higher energy uptake, which can be allocated to any function (van Noordwijk and de Jong, 1986). However, when food is scarce, specific functions may be prioritized over others. In lecithotrophic live-bearing animals, where all resources are allocated to the eggs prior to fertilization, adverse food conditions may reduce maternal growth, fat reserves and fecundity (Bashey, 2008; Reznick et al., 1996; Wang et al., 2017), yet also increase the relative investment in offspring size (Bashey, 2008; Reznick et al., 1996). In this example, mothers adaptively modified offspring phenotype in preparation for predicted, adverse environmental conditions (low food availability). This response is presumably advantageous, as under competitive adverse food conditions, mothers gain fitness benefits by producing larger offspring (Leips et al., 2013; Parker and Begon, 1986). However, as optimal offspring size is given by the offspring size–performance relationship, which depends on the environment, the advantage of producing larger offspring at birth diminishes or disappears in favourable environments (Bashey, 2008; Leips et al., 2013; Parker and Begon, 1986), and the predicted optimal strategy for the mother is to produce more numerous but smaller offspring (Jørgensen et al., 2011). If the maternal environment reliably predicts future environmental conditions, females may evolve the ability to adaptively adjust offspring phenotype at birth, based on environmental cues (Mousseau and Fox, 1998a).

The modulation and timing of nutrient acquisition and allocation also affect life-histories (Zera and Harshman, 2001). In matrotrophic live-bearing animals, experimental manipulations of food availability can impact the pattern of resource allocation to offspring (Banet and Reznick, 2008; Banet et al., 2010; Pollux and Reznick, 2011; Reznick et al., 1996; Van Dyke and Griffith, 2018). Instead of allocating all resources to the eggs prior to fertilization (i.e. lecithotrophy), matrotrophic species transfer nutrients to their embryos throughout gestation via a placenta (Pollux et al., 2014; Reznick et al., 2002; Wourms, 1981). Because matrotrophic species continuously supply embryos with resources, determination of brood size (i.e. number of embryos per brood) and offspring size are decoupled (Pollux and Reznick, 2011). When resource conditions during pregnancy suddenly deteriorate, matrotrophic species may not be able to optimally provision their embryos. Because females cannot abort embryos in response to food shortage and maternal fat reserves do not fully buffer females during gestation (Banet and Reznick, 2008; Banet et al., 2010; Pollux and Reznick, 2011; Reznick et al., 1996), sudden resource declines inevitably result in smaller, worse-conditioned (i.e. having lower fat reserves) offspring at birth. Small offspring size in low food conditions is associated with a lower survival (Bashey, 2008; Leips et al., 2013; Parker and Begon, 1986). Therefore, matrotrophy has been argued to be maladaptive in fluctuating resource environments (Pollux and Reznick, 2011; Reznick et al., 1996; Trexler and DeAngelis, 2003).

Differential resource allocation to offspring size at birth can impact postnatal development and survival (Mousseau and Fox, 1998b). In fish, body size is linked to locomotor performance (Gibb et al., 2006). Smaller offspring are likely to perform worse on fast-start escapes (Dial et al., 2016), presumably decreasing survival probability in environments with high predation risks. Prey-capture abilities immediately after birth also heavily depend on swimming performance. Newborn live-bearing fish are super-precocial, having functional prey-capture abilities at birth and relying on active exogenous feeding after birth (Lankheet et al., 2016). Moreover, they rapidly develop the visuo-motor skills required for prey capture during the first days after birth and effectively improve their success rate, promoting food uptake and survival (Lankheet et al., 2016). Offspring size thus affects locomotor performance and prey-capturing abilities after birth. However, it is still unknown to what extent restricted maternal food availability during pregnancy affects offspring swimming performance in fast-start escapes and in prey capture after birth.

Here, we examine the effects of maternal food restriction during pregnancy on growth and locomotor performance of offspring after birth in the matrotrophic fish species *Phalloptychus januarius* Hensel 1868 (family Poeciliidae). If matrotrophy is maladaptive under these circumstances, one may expect differences in offspring size as well as locomotor performance. Specifically, we measured: (i) size and body fat of offspring at different ages (i.e. 0 and 7 days), (ii) fast-start escape performance at birth, and (iii) swimming kinematics while feeding during the first week after birth, to quantify immediate and early postnatal effects of food restriction during pregnancy on offspring size, quality, and locomotor performance. As a proxy for locomotor performance, we focused on mean and maximum speed and acceleration of fast starts and feeding actions. By combining the different measurements, we reveal consequences of maternal food restriction for life-history variation and its implications for the quality and performance of newborn fish in a placental species.

## MATERIALS AND METHODS

### Experimental animals

*Phalloptychus januarius* is endemic to Brazil and is known from coastal drainages in Rio de Janeiro, São Paulo, and Paraná States of Brazil (Lucinda, 2005). The *P. januarius* used in this experiment were laboratory born and derived from laboratory stocks originally collected in the Rodrigo de Freitas Lagoon, Rio de Janeiro (Brazil) in November 2006 and held at the Pollux lab (Wageningen University, the Netherlands). In the Rodrigo de Freitas lagoon, *P. januarius* co-occurs with a variety of piscivorous fish (Andreata, 2012), birds (Santi et al., 2020) and bats (Luz et al., 2011), which collectively may represent a predation risk. Moreover, in their natural habitat they may experience both intra- and interspecies competition for food.

All procedures were approved by the Animal Ethics Committee of Wageningen University and Research (permit number 2018.W-0022.002).

### Maternal food treatment during gestation

The timing and length of maternal food restriction during (and prior to) pregnancy may influence offspring development. To test how, we conducted a 7-week experiment in 2019 to study the effects of maternal resource restriction during pregnancy on offspring. Females received *ad libitum* food for 1 week and were then given either a ‘low-food’ (LF) or ‘high-food’ (HF) ration for 6 weeks. Offspring born during the first week of the experiment served as a control, since they did not suffer from maternal food restriction. Offspring born during the second week were (indirectly) ‘exposed’ to maternal food restriction only during the last 25% of their development (embryonic development in *P. januarius* takes approximately 4 weeks until birth; Pollux and Reznick, 2011). Offspring born during the 3rd week were exposed during the second half of their development and offspring born during the 4th week during the last 75% of their development. Offspring born during week 5*–*7 were exposed during 100% of their development, but differed in the length of additional maternal food restriction prior to the start of their development: i.e. 0 weeks for offspring born during the fifth week to 2 weeks for offspring born during the seventh week.

Prior to the experiment, we set up 50 8-litre aquaria, each containing one mature male and female *Phalloptychus januarius*. This is a particularly interesting study species within the context of this study, because it has superfetation (Pollux et al., 2009): the ability to carry 7 to 14 temporally overlapping broods at different developmental stages. This means that over the course of the experiment each female will give birth to offspring that have been exposed to maternal food restriction during a different developmental period and for a different length of time. All fish were fed *ad libitum* prior to the start of the experiment, at which time the males were removed. Females were re-mated once overnight, after 3 weeks, to ensure a sufficient supply of sperm. There was a fourfold difference in food quantity between the LF and HF treatments, consisting of either 25 μl (LF) or 100 μl (HF) liver paste in the morning and 25 μl or 100 μl of newly hatched brine shrimp (*Artemia salina*) in the afternoon. Previous experiments indicated that the LF rations were sufficient to sustain reproduction while the HF rations were close to *ad libitum* feeding (Pollux and Reznick, 2011). We measured female body length (to the nearest mm) and wet mass (to the nearest 0.1 mg) at the beginning of the food treatment (i.e. after week 1) and at the end of the experiment (i.e. after week 7).

### Collection of offspring for various measurements

Experimental tanks were checked daily during the 7-week experiment for the presence of newborn offspring. These offspring were subsequently used for various experimental procedures: one randomly selected subset of offspring was used to measure dry mass and body fat (on day 0 and 7 after birth) using established protocols (Supplementary Materials and Methods 1). A second subset was used to quantify the fast-start escape performance (on day 0) and swimming kinematics during feeding (on day 0, 2 and 7 after birth) (see below for more details).

### Locomotor performance of offspring

Individual fish were isolated in Petri dishes (Ø 55 mm) and stored in an incubator at 24°C. The fish were subsequently transferred to an experimental set-up to record either the fast-start escape performance on day 0 or the swimming kinematics during feeding on day 0, 2 and 7 after birth. The fish were filmed from the dorsal side against an array of LEDs behind a white, translucent plate using a high-speed video camera (Supplementary Materials and Methods 2). The water level was kept at 5 mm to minimize vertical movements of the fish (average body length of newborn offspring: ∼7 mm). Following the performance measurements, the fish were euthanized and preserved to measure dry mass and fat content (Supplementary Materials and Methods 1).

#### Fast-start escape response

Throughout the experiment, up to 15 offspring were collected each Monday and Thursday (if available) to measure escape performance at birth. For this, the fish were transferred to smaller Petri dishes (Ø 35 mm). A maximum of 5 dishes, each containing a single fish, were simultaneously filmed. The fast-start escape manoeuvre was initiated after a 5 min acclimation period by dropping a weight on the plate. Fish were allowed to recover for 5 min, before initiating a second and third fast start.

#### Swimming kinematics during feeding

**Fig. 1.**
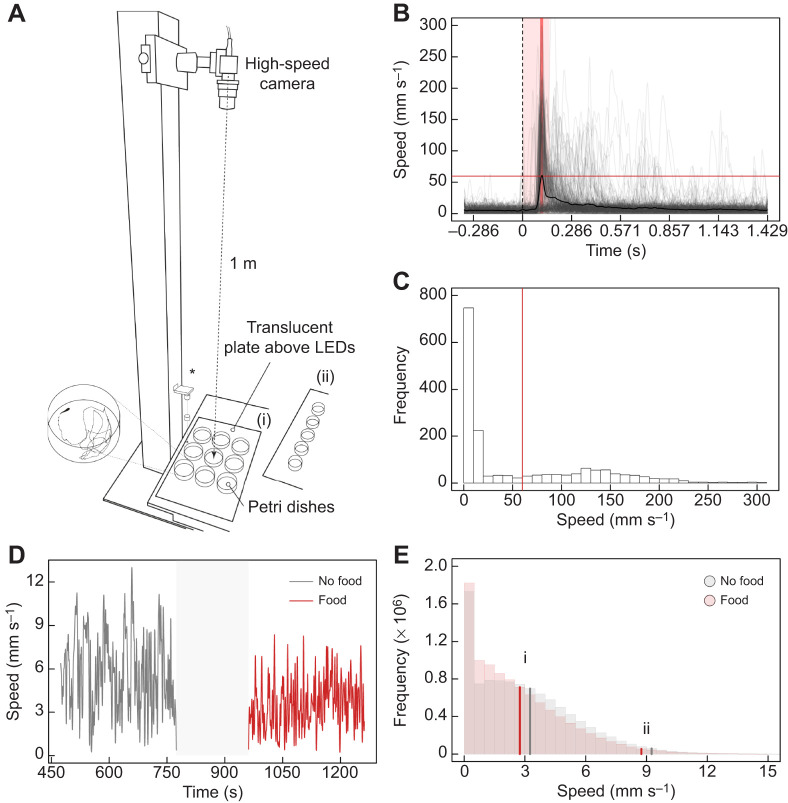
**Experimental set-up to record swimming kinematics during feeding and fast-start escape performance.** (A) Fish were filmed at high speed from the dorsal side. For (i) a maximum of 9 Petri dishes, each with a single fish, were placed on pre-defined positions on the plate (arranged in a 3×3 pattern). For (ii), a maximum of 5 dishes were placed on the plate (arranged in a 1×5 pattern). Fast-start escapes were initiated by dropping a weight on the plate (*). (B) Speed profiles of all fast-start escapes (*n*=270 fast starts). Dashed vertical line indicates the time at which the weight was released to trigger an escape maneuver. Solid black curve shows the overall mean speed. Mean and maximum performance parameters were extracted from a period that was sufficiently long to perform a fast start (light red rectangle). Fish were considered as having ‘responded’ if their speed during a 7 frame window around the maximum of the overall mean speed (thick red line) exceeded the threshold of 60 mm s^−1^ (thin red line). (C) Histogram of all instant fast-start speeds observed in the light red rectangle shown in B. The histogram shows two peaks, which correspond to individuals that have either responded to the stimulus or not. The response threshold for a fast start (thin red line in B and C) was defined as the speed observed at the minimum frequency in between the two peaks (60 mm s^−1^). (D) Example time trace of speed during an individual measurement of swimming kinematics while feeding. Mean and maximum locomotor performance parameters were extracted from the 5 min control period (grey line; no food available), and 5 min with available food (red line). The period during which food was supplied (grey rectangle) was filtered out to remove disturbances due to the experimenter. (E) Histogram of all instant speeds observed during the measurements of swimming kinematics while feeding. Grey: 5 min control period (no food present; *n*=162 fish); red: 5 min feeding period (*n*=161 fish). Solid lines correspond to the (i) overall mean speed and (ii) mean of speeds above 95% quantile.

Throughout the experiment up to 18 offspring were collected each Tuesday and Wednesday (if available) to measure swimming kinematics during feeding. The fish were held in the incubator at a 12 h light:12 h dark cycle for 1 week and fed daily *ad libitum* with newly hatched *Artemia.* Swimming kinematics during feeding were measured on day 0, 2 and 7 after birth. A maximum of 9 dishes, each containing a single fish, were simultaneously filmed (Fig. 1A). After an initial 5 min acclimation period, the swimming kinematics were first recorded for 5 min without food present (control). Subsequently, ∼30 newly hatched *Artemia* were added to each Petri dish to trigger feeding responses. The swimming kinematics were recorded for another 5 min. To sufficiently motivate offspring to feed, there was no additional food supplied prior to the feeding trials. The resolution in our videos was too low to track individual *Artemia*, therefore we lack information about prey-capture success of individual fish.

#### Video analysis

We used an in-house developed Python program to track the fish's silhouette in real-time and to automatically extract the location of the center of mass through time. Using the fish's position, we calculated swimming speed and linear acceleration (Supplementary Materials and Methods 3). As a proxy for locomotor performance, we extracted the mean and maximum speed and acceleration for each fast start and food response. For fast starts, we used a period after releasing the weight, which was sufficiently long to capture the response (0*–*0.157 s; Fig. 1B). Response intervals thus included a baseline during the time it took for the weight to hit the plate. Speed profiles were considered as fast-start responses if the speed during a 7 frame (0.103*–*0.120 s) window around the maximum of the overall mean speed exceeded the threshold of 60 mm s^−1^ (Fig. 1B,C). For food responses, we used the 5 min control period (no *Artemia* present), as well as the 5 min feeding period (Fig. 1D). Because extreme values for speed and acceleration are relatively sensitive to measurement noise, we defined the maximum speed and acceleration as the mean of the values above the 95% quantile, rather than the actual maximum values (Fig. 1E).

### Statistical analysis

All estimations were carried out in a Bayesian framework using the MCMCglmm package (https://CRAN.R-project.org/package=MCMCglmm; Hadfield, 2010) in R v. 3.5 (https://www.r-project.org/). Multivariate models allowed for the covariance between the residuals of all responses. Convergence was assessed by visual examination of the traces and the autocorrelations of the parameter chain was checked to be less than 0.1.

To identify potential effects of maternal food restriction on maternal wet mass and standard length, each trait was fitted in a univariate LMM. Fixed effects included treatment (LF or HF), experimental day (9 or 51), as well as treatment×day. In the case of maternal wet mass, maternal standard length was fitted as an additional covariate to quantify mass changes relative to length. Maternal identity was fitted as random intercept to correct for maternal variance sources not accounted for by the fixed effects (Hagmayer et al., 2018). Females that were not pregnant at the end of the experiment (*n*=2) or that died before (*n*=3), were excluded from the analysis. In addition, maternal fecundity was fitted in a GLMM using a log link for the Poisson-distributed response. Fixed effects included treatment, experimental week (1*–*7), as well as treatment×week. Maternal identity was fitted as random intercept (see above).

The effects of maternal food restriction on offspring phenotypic traits (dry mass, lean mass and body fat) were analysed by fitting all traits in a multivariate LMM as a function of treatment (LF or HF). Additional fixed effects included experimental day (day), day^2^, age (0 or 7 days), treatment×day, and treatment×age. Another fixed effect specified whether the offspring were found alive or dead. The probability of finding alive offspring significantly decreased throughout the experiment (β_post.mean_=−0.065, *P*_MCMC_=0.001), but did not differ between food treatments (β_post.mean_=0.008, *P*_MCMC_=0.732). The cause of the increasing frequency of dead offspring is unclear and warrants further research. Maternal identity was fitted as random intercept (see above). To optimize normality and homoscedasticity of the model residuals, body fat was square-root transformed.

For fast-start escapes we first analysed the probability of an individual to ‘respond’, which is used as a proxy for the behavioural propensity to react to the startle stimulus. To model the potential effects of treatment, the individual's response (yes or no) was fitted as a function of treatment (LF or HF), experimental day, as well as treatment×day in a GLMM using a logit link for the binomial-distributed response. Secondly, the mean and maximum speed and acceleration (all ln-transformed) of identified responses were fitted in a multivariate LMM. Fixed effects were treatment, experimental day, as well as treatment×day. In both models, maternal identity and Petri dish position were fitted as random intercepts. The latter accounts for potential effects of the Petri dish position relative to the camera and stimulus (i.e. location of weight drop). Moreover, offspring and replicate trial identity were fitted as random intercepts to account for pseudo-replication and for variation through habituation to the stimulus, respectively.

Likewise, the effects of maternal food restriction on swimming kinematics during feeding were analysed by fitting the mean and maximum speed and acceleration (all ln-transformed) in a multivariate LMM. To specifically quantify the effect of food supply, the model was fitted to the locomotor performance parameter extracted during: (i) the 5 min control period (no food supply) and (ii) the 5 min feeding period. To reduce model complexity, data from the two periods were analysed in separate models. Fixed effects included treatment (LF or HF), experimental day, age (0, 2 or 7 days), treatment×day and treatment×age. Maternal and offspring identity, as well as Petri dish position were fitted as random intercepts (see above).

## RESULTS

### Maternal length, wet mass, and fecundity

At the beginning of the food treatment, maternal standard length (SL) did not significantly differ between LF and HF females (β_post.mean_=−0.615, *P*_MCMC_=0.296). However, maternal wet mass was significantly lower for a given length in HF females (β_post.mean_=−0.029, *P*_MCMC_=0.008; Fig. S1). Over the course of the experiment, LF and HF females both showed an increase in SL (Fig. S1A). LF females, however, lost significantly more mass for a given length compared with HF females (0.08 vs. 0.03 g; *P*_MCMC_=0.004; Fig. S1B). Finally, maternal fecundity did not significantly change throughout the experiment (β_post.mean_=0.021, *P*_MCMC_=0.374) and did not differ between food treatments (β_post.mean_=0.006, *P*_MCMC_=0.882).

### Offspring size and body composition at birth

**Fig. 2.**
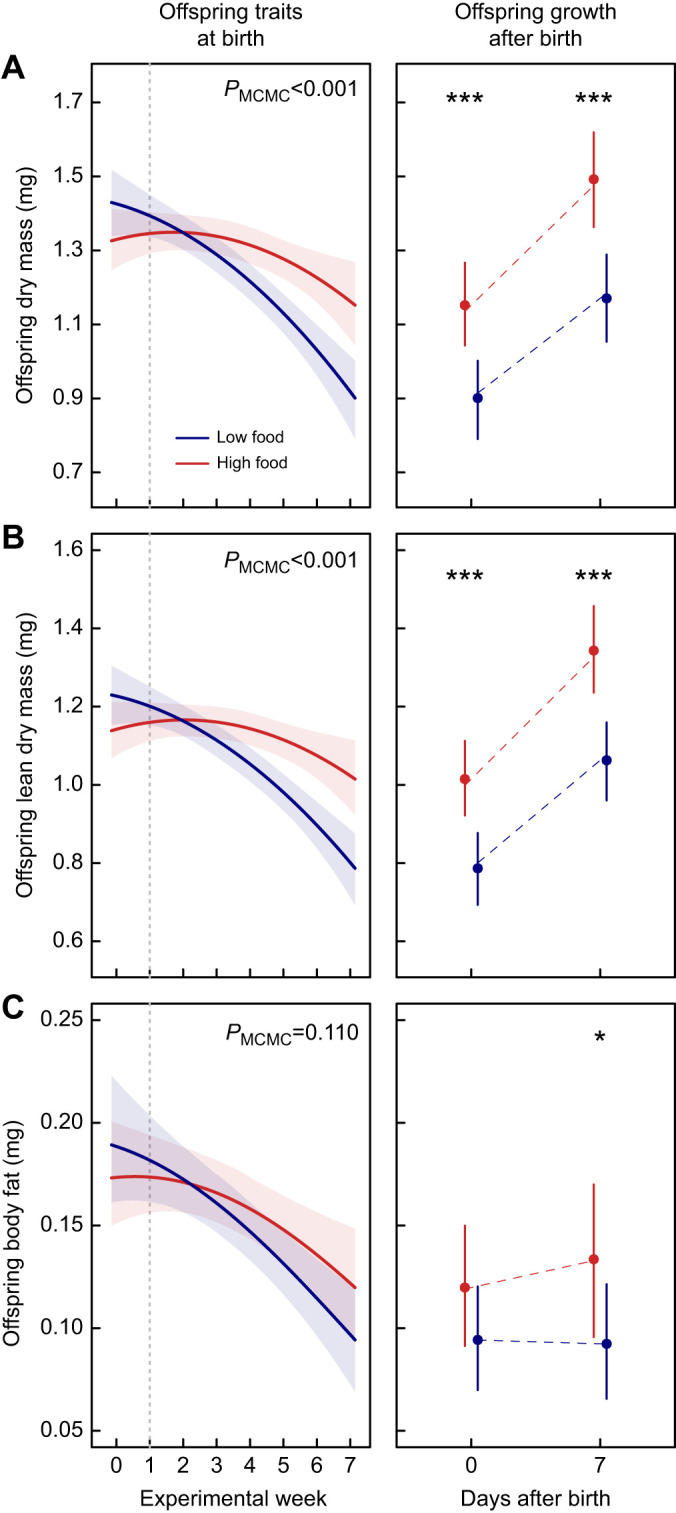
**The effect of maternal food treatment in *Phalloptychus januarius* during pregnancy on offspring phenotypic traits.** (A) Dry mass, (B) lean dry mass and (C) body fat (±95% CI) (*n*_LF_=300, *n*_HF_=287). Left panels in A–C show effect of the length of maternal food treatment during pregnancy on offspring traits at birth for offspring born during the 7-week experiment [in the first week, all females received *ad libitum* food, the dashed vertical line indicates the start of the 6-week food treatment; blue: low food (LF); red: high food (HF)]. *P*_MCMC_-values for the interaction between experimental day and treatment are given at the top. Right panels in A–C show increase in offspring dry mass, lean dry mass and body fat during the first week after birth, predicted for offspring that were born at the end of the 7-week experiment for both food treatments. Estimates are based on fish that were held in the laboratory for 1 week to measure swimming kinematics during feeding. Dashed lines represent linear fits throughout the posterior samples of a given food treatment. ***P_MCMC_≤0.001, *P_MCMC_≤0.05, *P*_MCMC_>0.05, n.s.

The dry mass and lean mass of offspring at birth significantly decreased throughout the 6-week food treatment in both LF (dry mass: β_post.mean_=−0.010, *P*_MCMC_<0.001; lean mass: β_post.mean_=−0.008, *P*_MCMC_<0.001) and HF females (dry mass: β_post.mean_=−0.003, *P*_MCMC_=0.012; lean mass: β_post.mean_=−0.002, *P*_MCMC_=0.038); however, this decrease was stronger in LF females (Fig. 2A,B, left panels). As a result, the offspring born at the end of the experiment (i.e. experimental week 7) were significantly lighter and leaner for LF than HF females (dry mass: β_post.mean_=−0.251, *P*_MCMC_<0.001; lean mass: β_post.mean_=−0.228, *P*_MCMC_<0.001; Fig. 2A,B, left panels). Offspring body fat significantly decreased throughout the food treatment (β_post.mean_=−0.001, *P*_MCMC_=0.010; Fig. 2C, left panel), but did not differ between LF and HF females (β_post.mean_=−0.001, *P*_MCMC_=0.110).

### Offspring size and body composition 7 days after birth

Fish held in the laboratory for 1 week to measure swimming kinematics during feeding were additionally used to study growth after birth. The difference in dry mass, lean mass, and body fat at birth observed at the end of the experiment persisted during the first week after birth (Fig. 2A–C, right panels). The body fat of offspring from HF females slightly increased during the first week after birth relative to offspring from LF females, resulting in a significantly different body fat of one-week-old offspring (β_post.mean_=4.1×10^−2^, *P*_MCMC_=0.040; Fig. 2C, right panel).

### Offspring fast-start escape response at birth

**Fig. 3.**
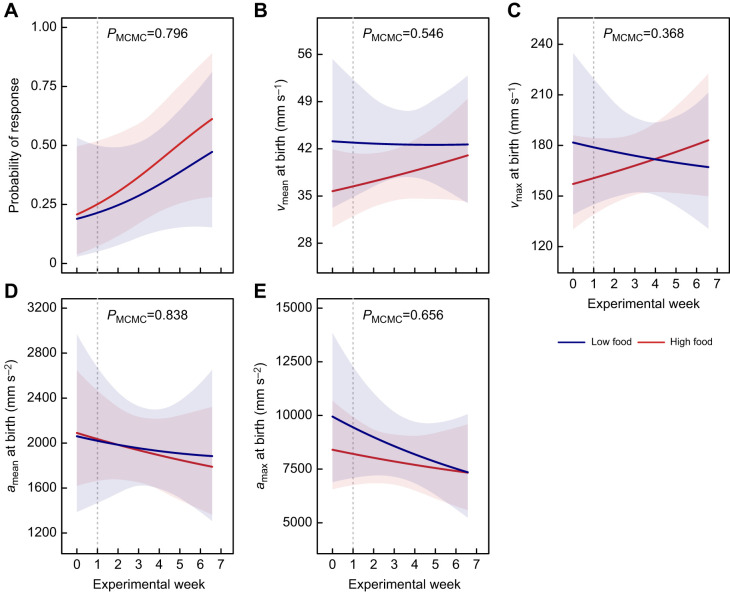
**The effect of the length of maternal food treatment in *P. januarius* during pregnancy on offspring fast-start performance at birth.** (A) Probability of newborn offspring response to the stimulus during fast-start trials (±95% CI) (*n*_LF_=94, *n*_HF_=113). The responsiveness is predicted for both food treatments (blue: low food; red: high food) and throughout the experiment. The dashed vertical line indicates the start of the 6-week food treatment. *P*_MCMC_-value for the interaction between experimental day and food treatment is given at the top. (B–E) Swimming performance of newborn offspring during fast-start escape response (*n*_LF_=24, *n*_HF_=31). During the 7-week experiment, newborns were collected weekly to assess 4 swimming kinematic parameters: mean speed (B), maximum speed (C), mean acceleration (D) and maximum acceleration (E) (±95% CI) at birth. These parameters are predicted for both food treatments (blue: low food; red: high food) and throughout the experiment. The dashed vertical line indicates the start of the 6-week food treatment. *P*_MCMC_-values for the interaction between experimental day and treatment are given at the top.

The probability of offspring responding to the stimulus tended to increase throughout the 7-week experiment, although not significantly (*β*_post.mean_=0.044, *P*_MCMC_=0.056). This increase was similar in both food treatments (*β*_post.mean_=−0.010, *P*_MCMC_=0.796; Fig. 3A). Likewise, the mean and maximum speed and acceleration during the fast start did not significantly change throughout the experiment, nor did it significantly differ between food treatments (Fig. 3B–E).

### Offspring swimming kinematics while feeding in the first week after birth

**Fig. 4.**
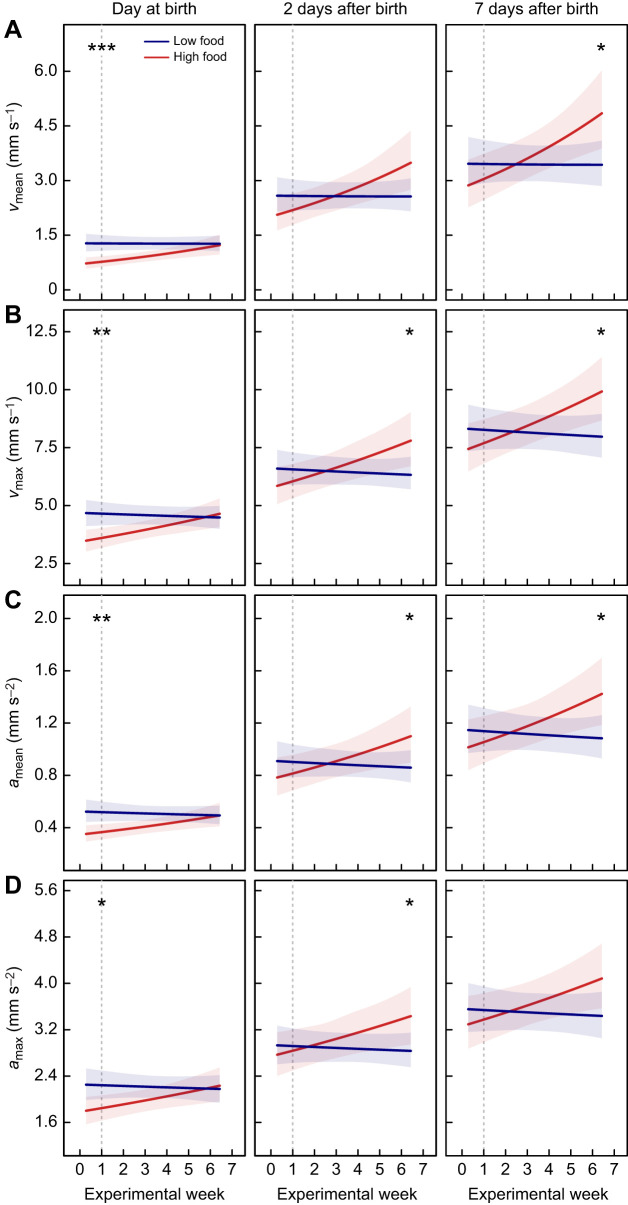
**The effect of the length of maternal food treatment in *P. januarius* during pregnancy on the swimming kinematics of offspring while feeding.** During the 7-week experiment, newborns were collected weekly to assess 4 swimming kinematic parameters: mean speed (A), maximum speed (B), mean acceleration (C) and maximum acceleration (D) (±95% CI). These parameters are predicted throughout the experiment for both food treatments (blue: low food; red: high food) at 3 different ages: at birth (0 days, left panels), 2 days (middle panels) and 7 days (right panels). The dashed vertical line indicates the start of the 6-week food treatment. Significance codes are given at the top for the difference between both food treatments at the start of the food treatment and at the end of the experiment. *n*_LF_=183, *n*_HF_=135; ***P_MCMC_≤0.001, **P_MCMC_<0.01, *P_MCMC_≤0.05, *P*_MCMC_>0.05, n.s.

Prior to the start of the food treatment (i.e. left of the dashed lines in Fig. 4), HF offspring showed a lower mean and maximum speed and acceleration at birth compared with LF offspring (Fig. 4A–D, left panels). As food availability had not been manipulated at this stage, this indicates a random bias in maternal, and hence offspring, phenotypes at the beginning of the experiment. This difference in swimming kinematics during feeding at birth diminished during the experiment. Maternal food restriction during pregnancy, therefore, had a significant effect on swimming kinematics during feeding. Specifically, the mean and maximum speed and acceleration did not significantly change throughout the experiment in LF offspring (*v*_mean_: β_post.mean_=−0.2×10^−3^, *P*_MCMC_=0.948; *v*_max_: β_post.mean_=−1.0×10^−3^, *P*_MCMC_=0.584; **a**_mean_: β_post.mean_=−1.3×10^−3^, *P*_MCMC_=0.576; **a**_max_: β_post.mean_=−0.8×10^−3^, *P*_MCMC_=0.686), but significantly increased throughout the experiment in HF offspring (*v*_mean_: β_post.mean_=0.012, *P*_MCMC_<0.001; *v*_max_: β_post.mean_=0.007, *P*_MCMC_=0.002; **a**_mean_: β_post.mean_=0.008, *P*_MCMC_=0.010; **a**_max_: β_post.mean_=0.005, *P*_MCMC_=0.034).

Furthermore, there was a clear ontogenetic effect on the swimming kinematics while feeding during the first week after birth, with 2-day-old (Fig. 4A–D, middle panels) and 7-day-old (Fig. 4A–D, right panels) offspring of both food treatments showing increasingly higher speed and acceleration than newborn offspring (Fig. 4A–D, left panels). This ontogenetic effect was further influenced by maternal food availability during pregnancy, with the locomotor performance of HF offspring improving relative to that of LF offspring. This resulted in significant differences in most, but not all, kinematic parameters between both food treatments in 1-week-old offspring born at the end of the experiment (*v*_mean_: β_post.mean_=1.414, *P*_MCMC_=0.028; *v*_max_: β_post.mean_=1.951, *P*_MCMC_=0.020; **a**_mean_: β_post.mean_=0.340, *P*_MCMC_=0.034; **a**_max_: β_post.mean_=0.646, *P*_MCMC_=0.058; Fig. 4A–D, right panels). Interestingly, the segregation in locomotor performance between LF and HF offspring throughout the experiment and during the first week after birth was only apparent when food was supplied: during the 5 min control period (i.e. before food was supplied), LF and HF offspring showed similar mean and maximum speed and acceleration (Fig. S2). This suggests that the length of maternal food restriction during pregnancy likely affects the feeding capabilities of offspring.

## DISCUSSION

We examined to what extent the length of maternal food restriction during pregnancy affects size, quality, and performance of offspring in the matrotrophic live-bearing fish species *P. januarius*. Females that were fed a low food ration (LF) during gestation produced offspring with significantly lower dry mass and lean dry mass with a tendency of having fewer fat reserves at birth compared with females fed with a high food ration (HF). The longer the maternal food treatment during (and prior to) pregnancy, the more pronounced were these phenotypic differences. Furthermore, these differences persisted (dry mass and lean dry mass), or became even more pronounced (body fat), during the first week after birth, suggesting slower postnatal body fat gain of the smaller offspring. Maternal food restriction during pregnancy did not impact the fast-start escape performance of offspring at birth; however, it did influence several swimming kinematic parameters while feeding during their first week after birth. Together, our findings show that maternal food restriction during pregnancy adversely affects offspring size, postnatal body fat gain, postnatal improvement of locomotor performance, and hence possibly their competitive abilities (Bashey, 2006) after birth.

### Effects of maternal food restriction on offspring fast-start escape performance at birth

The probability of offspring responding to a startle stimulus tended to increase over the course of the experiment, for both treatments. Fish may vary their neural threshold for triggering a fast-start response (Wakeling, 2006) depending on stress levels or health condition (Chick and Van Den Avyle, 2000). Fast escapes are energetically expensive and cannot be repeated at a high rate (Frith, 1990). The observed increase in response rate might thus reflect the modulation of a neuronal threshold, presumably in the Mauthner neurons that mediate the response, or in neurons that stimulate the Mauthner neurons. However, it is currently unclear why the probability to induce a startle response increased over the course of the experiment.

In studying the fast-start escape behaviour, we have at least partially accounted for a change in motivation or threshold by selecting only trials with a clear response to the stimulus. This is important because differences in motivation can introduce noise and variability in the response parameters (Losos et al., 2002). In general, the fast-start escape performance depends on physiological and mechanical muscle properties, as well as muscle activation and body form parameters (Fleuren et al., 2019; Wakeling, 2006). Larger fish typically achieve higher maximum velocities during fast starts owing to larger muscle mass and body length, and production of greater bending moments (Dial et al., 2016; Gibb et al., 2006; Voesenek et al., 2020; Wakeling, 2006). Since LF offspring are born significantly lighter and leaner at the end of the experiment compared with HF offspring, it is surprising that we do not find an effect on their fast-start escape performance (Dial et al., 2016). This may be due to a low statistical power to detect significant differences because our response selection removed about 67% of the data (Fig. 1B). Although additional analyses with different response thresholds indicate that these findings are relatively robust (Supplementary Materials and Methods 4; Figs S3–S5), we have to be cautious when concluding that maternal food restriction does not impact fast start escapes at birth. Future studies should try to maximize sample sizes to yield extended measurements of locomotor performance.

### Effects of maternal food restriction on offspring swimming kinematics during feeding

Newborn poeciliid fish are super-precocial, having functional prey-capture abilities at birth and relying on active exogenous feeding after birth (e.g. *Girardinus metallicus*; Lankheet et al., 2016). Their prey-capturing ability undergoes a rapid integrated development of the visuo–motor system in the first days after birth. Swimming speed and acceleration are key parameters determining prey-capture success rate (Lankheet et al., 2016). In *P. januarius*, these parameters improve rapidly after birth: 1-week-old offspring showed higher mean and maximum speeds and accelerations than newborn offspring, both in LF and HF offspring. However, the locomotor performance during feeding improved significantly faster in HF offspring compared with that of LF offspring, during the first week after birth. Interestingly, this difference in locomotor performance cannot be explained by differences in ontogenetic growth between HF and LF offspring, because (i) although HF offspring were larger at birth than LF offspring, they showed similar increases in size during the first week after birth, and (ii) the segregation in locomotor performance between LF and HF offspring was only apparent when food was supplied. This suggests that maternal food availability during pregnancy affects the postnatal maturation of tissues associated with locomotion during feeding, which may cause differently developing feeding capabilities after birth between LH and HF offspring.

We found that LF offspring are smaller at birth and tend to have less body fat. It is likely that they also differed in other morphological and physiological features that can influence their postnatal development of locomotor performance during feeding. For instance, smaller guppy offspring were shown to have a lower degree of skeletal ossification at birth, which is considered a proxy for internal maturity (Dial et al., 2016). Skeletal ossification can directly affect swimming performance (Dial et al., 2016), and may also influence postnatal development of locomotor performance. In addition, locomotor performance depends on muscle fibre type (Rome et al., 1988) that undergoes a distinct shift in composition after birth (Veggetti et al., 1993). Similarly, maximum body curvature during prey-capture was shown to change after birth (Lankheet et al., 2016). Particularly, an increase in muscle mass-specific power output induces a higher body curvature (Wakeling, 2006), and consequently improves prey-capture success rate if the motion control is sufficiently matured (Lankheet et al., 2016). Better prey-capturing success enables uptake of more energy (via feeding) per unit of time, which can be allocated to either growth or quality (e.g. body fat). It is thus possible that LF and HF offspring differ in various morphological or behavioural parameters at birth (e.g. degree of skeletal ossification, composition of muscle fibre type, or body curvature), which then develop differently in HF and LF fish after birth. The improved prey-capture success and increased efficiency of resource acquisition in HF offspring compared with LF offspring may also explain why HF offspring gained body fat during the first week after birth, but LF offspring did not.

### Compounding effects on offspring growth

Overall, our findings suggest that differential resource allocation of mothers to offspring may not only cause LF offspring to be born smaller than HF offspring, but also ‘worse-conditioned’, i.e. showing a slower postnatal body fat gain and an inhibition of postnatal improvement of swimming capabilities during feeding. Consequently, these smaller offspring are likely to have lower competitive abilities in a resource-limited environment, for two reasons: first, larger offspring that carry more fat reserves have a competitive advantage over smaller offspring if they are born in environments where resources are scarce, allowing them to survive for longer periods of time under low food conditions (Bashey, 2006, 2008; Parichy and Kaplan, 1992). Secondly, larger offspring have better swimming performance during feeding, which may translate to superior prey-capture abilities (Lankheet et al., 2016), and hence, faster postnatal body fat gain, compared with smaller offspring.

Compensatory growth could be an adaptive strategy for small offspring to fully or even overcompensate a smaller body size in response to increased food availability following a period of growth restriction (Auer et al., 2010; Metcalfe and Monaghan, 2001). However, although LF and HF offspring were fed *ad libitum* after birth, we observed no compensatory growth for LF offspring in *P. januarius* during the first week after birth. The poorer swimming performance during feeding of LF offspring compared with HF offspring, potentially made it more difficult for the smaller LF offspring to catch up in mass during the first week after birth. This may have further exacerbated the differences: rather than displaying catch-up growth to compensate for their smaller size at birth, LF offspring fall further behind despite *ad libitum* food availability after birth. Interestingly, however, these differences in size and body composition between LF and HF offspring had disappeared at sexual maturity (Supplementary Materials and Methods 5; Fig. S6), indicating that in the long-term, LF offspring may be able to (partly) compensate for their poor nutrition during gestation.

### Contrasting findings in non-placental live-bearing species

Non-placental (i.e. lecithotrophic) live-bearing females provide all resources required for embryo development as yolk, prior to fertilization. Therefore, brood size and offspring size are determined prior to fertilization based on prior food availability (Reznick et al., 1996). Rather than resulting in smaller, worse-conditioned offspring at birth, adverse food conditions were shown to reduce maternal growth, fat reserves, and fecundity, yet also increase the relative investment in offspring size (Bashey, 2008; Reznick et al., 1996; Riesch et al., 2016). Here, mothers adaptively modified offspring phenotype in preparation for adverse environmental conditions (i.e. low food availability). This response is presumably advantageous, as under competitive adverse food conditions, mothers gain fitness benefits by producing larger offspring (Leips et al., 2013; Molina-Moctezuma et al., 2020; Parker and Begon, 1986).

Placental females, however, continuously supply their developing embryos with nutrients throughout pregnancy. Consequently, the timing of determining brood size and offspring size are likely decoupled (Pollux and Reznick, 2011; Reznick et al., 1996). Whereas brood size is determined prior to fertilization based on current food availability, offspring size is affected by food availability after fertilization, throughout gestation (Pollux and Reznick, 2011; Reznick et al., 1996). As a result, when resource conditions suddenly deteriorate, placental species may not be able to optimally provision their embryos. In contrast to lecithotrophic species, therefore, placental species lack the possibility to adaptively adjust offspring phenotype in response to sudden reductions in food availability.

### Conclusion

Our study shows that matrotrophy in poeciliids may be a maladaptive strategy in unpredictable fluctuating resource environments, because sudden reductions in maternal food availability during pregnancy result in smaller offspring with slower postnatal body fat gain and an inhibition of postnatal improvement of swimming capabilities during feeding. Future studies should try to explicitly quantify the consequences of maternal food restriction on prey-capture success rate of offspring to better understand the role of compromised postnatal development of locomotion in shaping offspring growth and hence fitness.
